# Primary cutaneous adenoid cystic carcinoma arising in the axilla: a case report and literature review

**DOI:** 10.3389/fonc.2026.1942206

**Published:** 2026-09-18

**Authors:** Si Li, Fang Yang, Yangyang Jiang, Shuanghai Hu

**Affiliations:** 1 Department of Dermatology, Shenzhen People's Hospital (The Second Clinical Medical College, Jinan University; The First Affiliated Hospital, Southern University of Science and Technology), Shenzhen, Guangdong, China; 2 Department of Dermatology, South China Hospital, Medical School, Shenzhen University, Shenzhen, China; 3 Department of Dermatology, National Clinical Research Center for Infectious Diseases, The Third People's Hospital of Shenzhen, Shenzhen, China; 4 Department of Pathology, Shenzhen People's Hospital (The Second Clinical Medical College, Jinan University; The First Affiliated Hospital, Southern University of Science and Technology), Shenzhen, Guangdong, China

**Keywords:** case report, metastasis, primary cutaneous adenoid cystic carcinoma, prognosis, tumor diameter

## Abstract

Primary cutaneous adenoid cystic carcinoma (PCACC) is a rare cutaneous adnexal malignancy. Although its clinical course is generally considered more indolent than that of salivary gland-derived counterparts, PCACC exhibits a significant propensity for local recurrence and a notable potential for distant metastasis. Here, we report a rare case of PCACC arising in the right axilla. Based on a comprehensive literature review and statistical analysis of previously reported cases, our findings suggest that a tumor diameter of ≥ 2.0 cm may be associated with an increased risk of metastasis. Therefore, for patients with tumors exceeding 2.0 cm, we suggest that careful determination of surgical margins and comprehensive staging evaluations—including whole-body PET-CT, sentinel lymph node biopsy, and long-term postoperative follow-up—may be considered.

## Introduction

Primary cutaneous adenoid cystic carcinoma (PCACC) is an exceptionally rare malignant adnexal neoplasm that arises directly from the skin, most frequently from eccrine or apocrine sweat glands, with no identifiable primary salivary gland or visceral involvement (1). Although the clinical behavior of PCACC is generally considered more indolent than that of its salivary gland counterpart, it retains a marked propensity for local recurrence and carries a non-negligible risk of distant metastasis, most commonly to the lungs and regional lymph nodes (2, 3). Histologically, PCACC is characterized by cribriform, tubular, and solid growth patterns, often accompanied by perineural invasion—a feature frequently observed and closely correlated with local aggressiveness and recurrence (4). Owing to its rarity and considerable morphological overlap with other adnexal tumors, PCACC is frequently misdiagnosed prior to surgery, leading to delayed intervention and suboptimal initial management.

Herein, we present a rare case of PCACC arising in the right axilla. In addition, through a systematic literature review and statistical analysis of all previously documented cases, our findings suggest that a tumor diameter of ≥2.0 cm may be associated with an increased risk of distant metastasis. These observations imply that for patients presenting with lesions exceeding this size threshold, a more comprehensive staging evaluation—including whole-body PET-CT and sentinel lymph node biopsy—may be considered, and that rigorous, long-term postoperative surveillance should be implemented to facilitate early detection of recurrence or metastatic spread.

## Case report

A 37-year-old female presented with a history of a subcutaneous nodule in her right axilla, which she had incidentally discovered 10 years prior. Initially, the lesion was approximately the size of a soybean, did not protrude above the skin surface, and presented as a moderately firm, erythematous mass adherent to the surrounding tissues with restricted mobility. As it was asymptomatic—lacking pain, tenderness, or pruritus—the patient did not seek medical evaluation at that time. Over the subsequent decade, the mass exhibited slow but progressive enlargement and eventually became painful (**Figure 1**).

**Figure 1:**
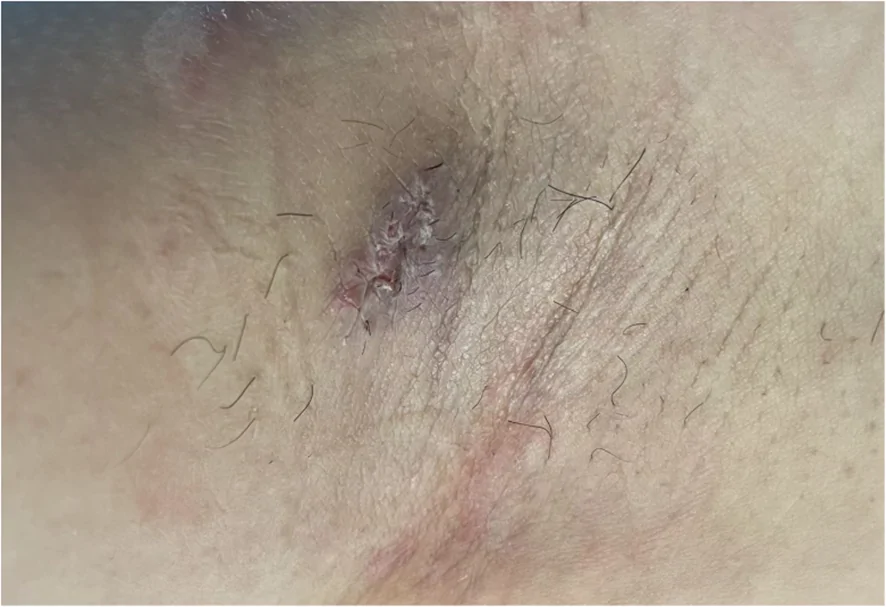
A 2.5 cm erythematous nodule with a roughened surface overlying the right axilla.

In November 2022, the patient presented to our dermatology outpatient clinic. Physical examination revealed a palpable, solitary erythematous nodule measuring approximately 2.5 × 2.0 cm in the right axilla. The lesion was firm, fixed to the underlying tissue, and tender on palpation, with overlying skin erythema but no ulceration or discharge. No palpable lymphadenopathy was detected in the ipsilateral axillary or supraclavicular regions. The initial clinical diagnosis was an epidermal cyst. After completing routine preoperative investigations, the patient underwent excision of the lesion with a planned lateral margin of 0.5 cm beyond the visible tumor border. Intraoperative frozen section margin assessment was not performed. Final pathological margin assessment revealed positive margins.

Postoperative histopathological examination revealed an adenoid cystic carcinoma with positive surgical margins. Microscopically, the tumor displayed a predominantly cribriform growth pattern. The neoplastic cells displayed marked pleomorphism and atypia, characterized by scant cytoplasm and large, hyperchromatic nuclei. The surrounding stroma was dense, and the tumor demonstrated infiltrative growth into the adjacent adipose tissue. Perineural invasion was not identified in the resection specimen. Lymphovascular invasion was not identified(**Figure 2A**). Immunohistochemical analysis revealed that the tumor cells were strongly positive for CK7, CD117, EMA, and SOX10, with focal positivity for S-100. The Ki-67 proliferation index was approximately 5% (**Figure 2B**). Based on these histomorphological features and the immunophenotype, a diagnosis of cutaneous adenoid cystic carcinoma was established.

**Figure 2:**
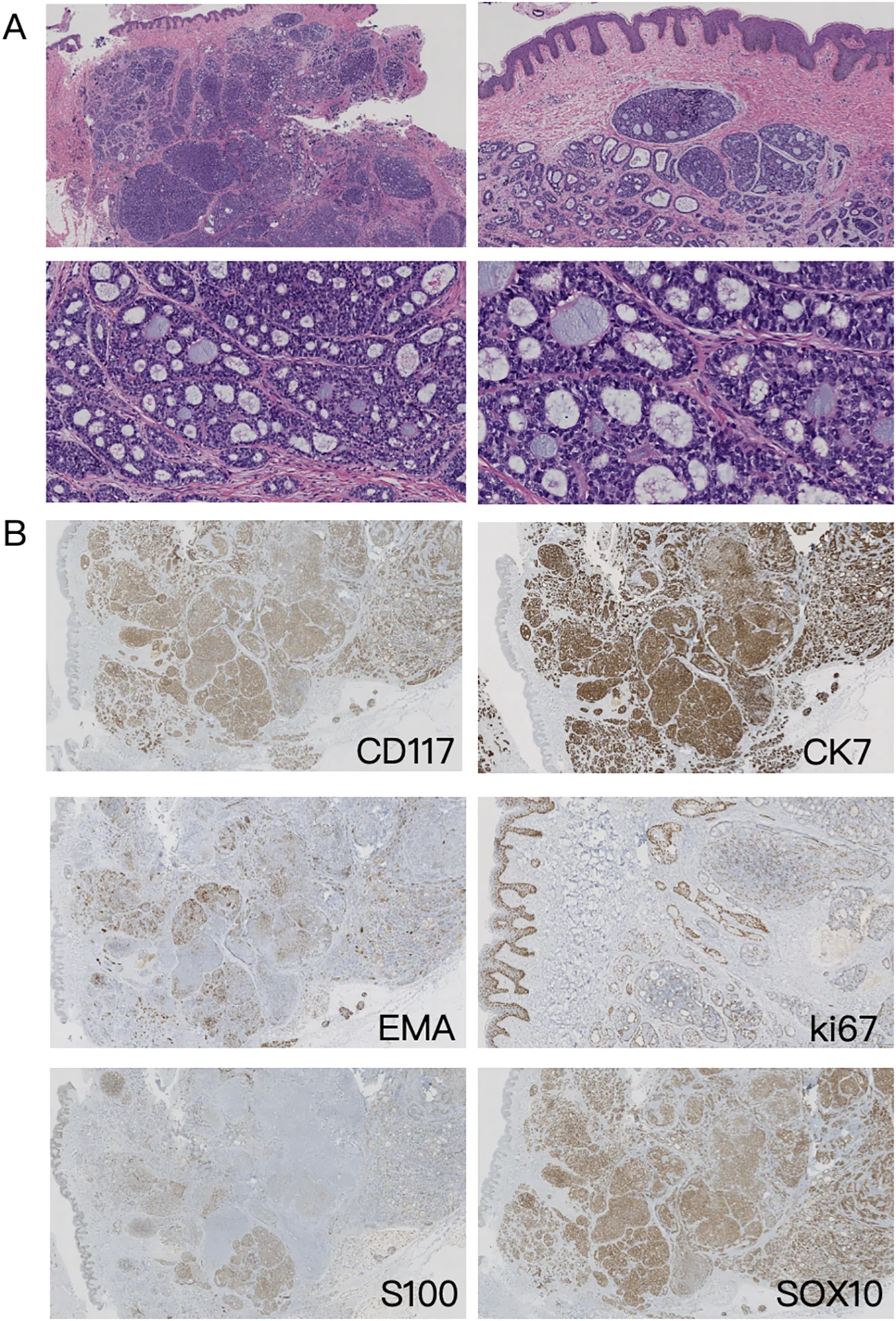
**(A)** Histological features (H&E 4×,10×, 20×, 40×) showing a circumscribed neoplasm composed of clusters of basophilic cells with a cribriform pattern in the dermo-hypodermal region, The tumor tissue exhibited tubular, cribriform, and solid growth patterns. The neoplastic cells varied in size and shape, displaying marked atypia, scant cytoplasm, and large, hyperchromatic nuclei. The stroma was dense, and the tumor had infiltrated the surrounding adipose tissue. Perineural invasion was not identified in the resection specimen. Lymphovascular invasion was not identified. **(B)** Immunohistochemistry plate (4×) showing the tumor cells were positive for CK7, EMA, CD117, and SOX10, with focal positivity for S-100 and a Ki-67 proliferation index of approximately 5%.

To exclude a metastatic origin from a primary salivary gland tumor, a whole-body PET-CT scan was performed. The imaging revealed a small, ill-defined patchy shadow in the subcutaneous tissue of the right axillary surgical bed, measuring approximately 7.4 mm × 6.6 mm, with mild 18F-FDG uptake (**Supplementary Material 1**). Histopathological examination and immunophenotyping confirmed adenoid cystic differentiation. PET-CT, combined with targeted clinical evaluation, revealed no extracutaneous primary tumor or distant metastasis, thereby supporting the classification of this lesion as primary cutaneous ACC in the appropriate clinicopathological context. The patient was counseled regarding the malignant nature of the tumor and the positive surgical margins, and a wide local excision was strongly recommended. The patient received detailed information regarding the histopathological diagnosis and the positive surgical margin, and the rationale for re-excision to achieve clear margins was thoroughly explained. She had independently consulted relevant medical literature and understood PCACC to be an indolent cutaneous tumor; initially, she therefore opted to defer further surgery, motivated by concerns about wound healing and the potential impact on her daily work, particularly because the lesion had caused no symptoms. Regular dermatology follow-up was recommended, with instruction to return if a subcutaneous mass becomes palpable in the axilla.

Three months postoperatively, the patient noted the emergence of a new subcutaneous mass at the surgical incision site. She returned for follow-up in early 2024. Physical examination revealed an approximately 2.5 cm postoperative scar in the right axilla, beneath which a palpable, firm subcutaneous mass measuring approximately 1.0 cm in diameter was identified. The overlying skin appeared unremarkable, with no evidence of exudation, purulence, or bleeding. Color Doppler ultrasonography demonstrated a well-circumscribed, hypoechoic mass measuring approximately 10 mm in the right axilla. The mass exhibited heterogeneous internal echogenicity and abundant internal arteriovenous blood flow signals, without evidence of invasion into the underlying muscle layer. Before the second surgery in March 2024, the patient underwent chest computed tomography (CT), color Doppler ultrasonography of the superficial mass, and ultrasonography of the axillary and cervical lymph nodes. No evidence of regional lymph node involvement or distant metastasis was identified.

In March 2024, the patient underwent a wide local excision of the recurrent right axillary tumor, followed by local flap reconstruction under local anesthesia. Intraoperatively, a spindle-shaped incision was designed with a 3.0 cm clinical margin extending beyond the palpable tumor, and the resection was carried down to the level of the deep fascia. No muscular invasion was observed. Intraoperative frozen section analysis confirmed clear surgical margins with no residual tumor. The postoperative wound healed uneventfully. The patient remained free of clinically and radiologically detectable recurrence or metastasis at 2 years after definitive re-excision (**Figure 3**).

**Figure 3:**
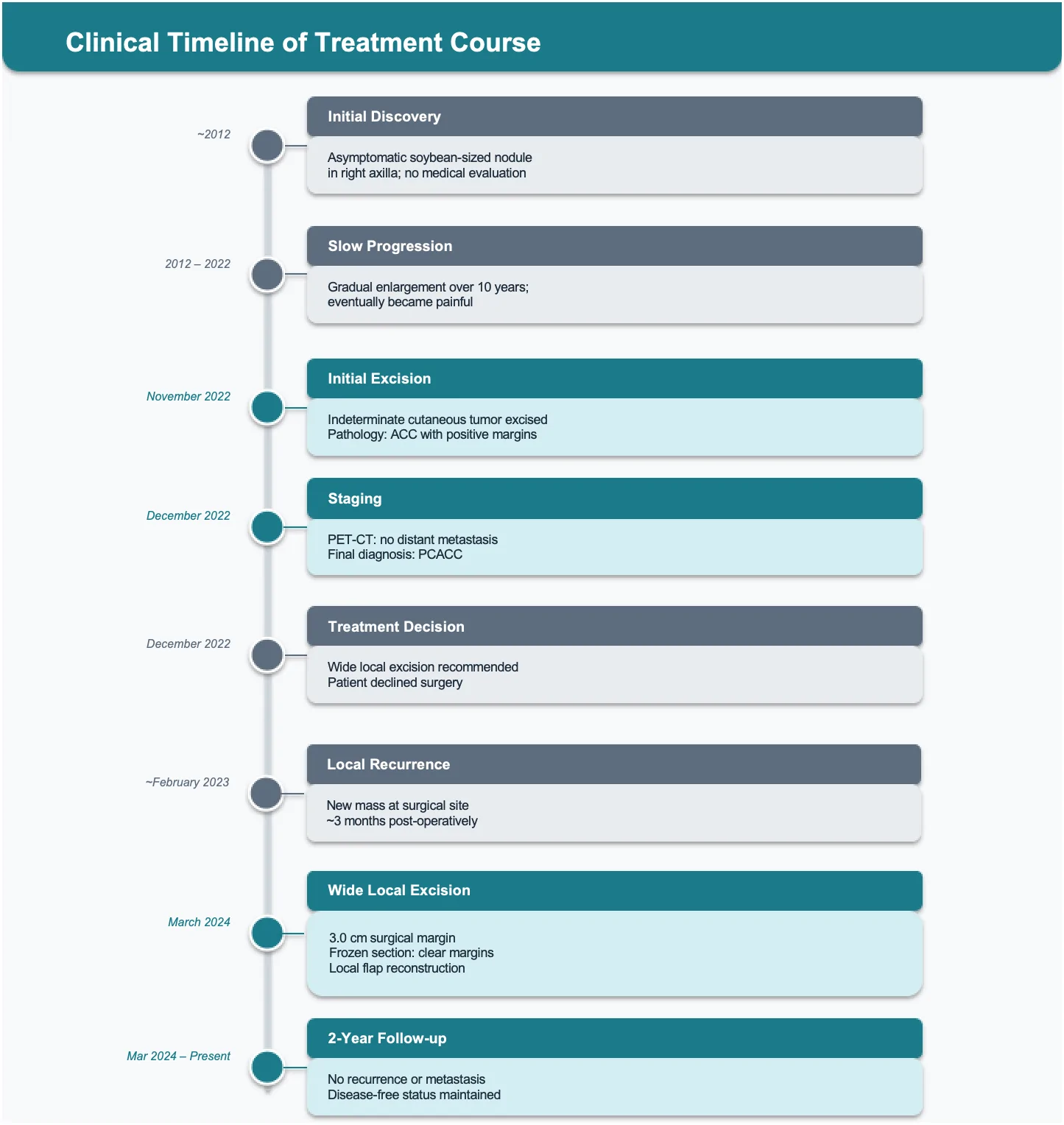
Clinical timeline of lesion progression, diagnostics, surgery, and short-term follow-up.

## Discussion

Primary cutaneous adenoid cystic carcinoma (PCACC) is an exceptionally rare cutaneous malignancy of glandular epithelial origin (5). Adenoid cystic carcinoma (ACC) most frequently arises from the major and minor salivary glands, as well as the glandular epithelium of the respiratory tract. The minor salivary glands and the submandibular gland are the most commonly affected sites, with approximately 40% of all ACC cases occurring in the head and neck region (6). In the skin, PCACC is postulated to originate from ceruminous glands, ductal epithelium, or myoepithelial cells, with eccrine sweat glands being the predominant source of cutaneous involvement (7). Furthermore, some investigators have proposed that PCACC may arise from the malignant transformation of ectopic parotid gland remnants. In the present case, the patient had a history of presenting to the Department of Oral and Maxillofacial Surgery for facial swelling and dental pain. Consequently, a whole-body PET-CT scan was crucial to definitively exclude the possibility of cutaneous metastasis from an occult primary tumor in the parotid, submandibular, or other salivary glands.

The clinical course of PCACC is typically insidious and protracted. The lack of specific clinical manifestations often leads to delayed diagnosis or misdiagnosis, with reported disease durations ranging from 1 to 30 years prior to definitive identification (3). Clinically, PCACC presents as a firm, ill-defined, slow-growing nodule that may protrude from the skin surface, often exhibiting a dark purple or erythematous hue. Due to its morphological similarity to other benign superficial soft tissue tumors, definitive diagnosis relies heavily on histopathological and immunohistochemical evaluation (3). In our case, the index lesion initially presented as a painless erythematous nodule, and the patient only sought medical attention when the nodule progressively enlarged and became painful. A subsequent wide local excision performed one year later achieved clear histological margins, and the patient remained free of clinically and radiologically detectable recurrence or metastasis at 2 years after definitive re-excision. This represents short-term follow-up, and prolonged surveillance remains necessary because PCACC can recur or metastasize after extended intervals.

Histologically, PCACC is composed of cribriform and tubular aggregates of basaloid cells in the dermis, often accompanied by true bilayered ducts and mucin-containing pseudocysts (8). The nuclei are characteristically hyperchromatic and compact with inconspicuous nucleoli, and mitotic figures are infrequent. The cytoplasm is pale-staining, and linear or globular deposits of eosinophilic material are frequently observed along the cell periphery. The tumor forms adenoid structures and small islands of cells, with glandular spaces often filled with mucin and lined by flattened epithelial cells. While the histological features of cutaneous ACC are generally distinctive, an accurate diagnosis necessitates a comprehensive immunohistochemical panel encompassing both epithelial and myoepithelial markers, supplemented by mucin histochemistry, to differentiate it from other adnexal neoplasms (9).Clinically, PCACC typically manifests as a slow-growing, firm dermal or subcutaneous nodule, most commonly affecting the scalp and the head and neck region. Histologically, the tumor is characterized by an infiltrative growth pattern with tubular, cribriform, and/or solid architecture, accompanied by true bilayered ducts and pseudocysts containing basophilic mucinous or hyaline basement membrane-like material. The presence of a biphasic ductal–myoepithelial cell population and perineural invasion constitutes important diagnostic clues (3, 10).

Microcystic adnexal carcinoma (MAC) represents a key differential diagnosis, particularly for infiltrative lesions in the head and neck area. MAC is typically composed of deeply infiltrative bland cords, keratin cysts, and syringomatous ducts embedded within a dense desmoplastic stroma. Although perineural invasion may be observed in both MAC and PCACC, the presence of keratin cysts, sclerotic stroma, and the absence of a prominent cribriform pattern with pseudocysts favor the diagnosis of MAC. Immunohistochemically, MAC frequently expresses CK5/6, CK15, CK17, and p63, with ductal structures highlighted by EMA and CEA. In contrast, PCACC exhibits a more characteristic biphasic immunophenotype, in which CK7, EMA, CEA, and CD117/c-KIT mark the luminal/ductal cells, while p63, SMA, calponin, SOX10, and variably S100 highlight the peripheral myoepithelial component (3, 10, 11).

Other sweat gland carcinomas—including syringoid eccrine carcinoma and poorly differentiated sweat gland carcinoma—may exhibit ductal differentiation and infiltrative growth, but generally lack the cribriform/tubular architecture, true bilayered ducts, and coordinated epithelial–myoepithelial immunophenotype that are hallmarks of PCACC. Eccrine porocarcinoma is more likely to demonstrate an epidermal connection, poroma *in situ* or residual poroma, marked cytological atypia, brisk mitotic activity, and necrosis. Hidradenocarcinoma tends to be more heterogeneous, comprising clear, squamoid, oncocytic, or mucinous cells, with variable expression of CK7, EMA, CEA, AR, EGFR, or HER2. These features contrast with the relatively monomorphic basaloid appearance and dual-cell population characteristic of PCACC (11, 12). The differential diagnosis of PCACC includes aggressive eccrine ductal neoplasms, such as squamoid eccrine ductal carcinoma and syringoid eccrine carcinoma, which may show deep infiltration, duct formation, squamoid differentiation, and perineural invasion. These tumors are often distinguished from PCACC by their prominent squamoid component, irregular ducts, marked atypia, and lack of the characteristic cribriform/tubular biphasic ductal–myoepithelial pattern. Appropriate clinicopathological correlation and a directed immunohistochemical panel are essential: in PCACC, CK7, EMA, CEA, and CD117/c-KIT support luminal differentiation, whereas p63, SMA, calponin, SOX10, and variably S100 support the peripheral myoepithelial component (13).

Finally, metastatic ACC or direct extension from a primary salivary gland tumor must be rigorously excluded. Salivary ACC and PCACC are often morphologically indistinguishable and share the expression of CD117, SOX10, p63, SMA, EMA, and CEA. Therefore, clinical history and appropriate imaging studies remain essential to confirm a primary cutaneous origin. In the present case, the characteristic cribriform/tubular morphology, mucinous pseudolumina, CD117 positivity, and EMA-supported ductal differentiation supported the diagnosis of ACC; however, the final diagnosis of PCACC was established only after clinicoradiological exclusion of an extracutaneous primary lesion. Notably, because S100 and DOG-1 expression may be variable, their negativity does not rule out PCACC (3, 10, 14).

Although PCACC is generally classified as a low-grade malignancy, it exhibits a well-documented propensity for perineural invasion, visceral involvement, and distant metastasis (15). Therefore, wide local excision with adequate safety margins remains the universally recommended cornerstone of treatment (7). For younger patients, a more aggressive surgical approach with expanded resection margins should be considered. The indication for regional lymph node dissection depends on the individual clinical scenario and is strictly warranted in cases with confirmed regional lymph node involvement (16). In the present case, given the absence of perineural invasion, visceral involvement, or lymph node metastasis, a wide local excision was performed. Intraoperative frozen section analysis was utilized to ensure complete tumor clearance and minimize the risk of local residual disease. PCACC is generally considered radio- and chemo-resistant; the decision to administer adjuvant chemoradiotherapy is typically guided by factors such as tumor size, anatomical location, depth of invasion, and margin status, although definitive evidence-based guidelines are currently lacking. Given that previous reports indicate the interval from surgical excision to recurrence can range from 1 to 40 years (17), long-term, rigorous clinical surveillance is mandatory for this patient.

Due to the extreme rarity of PCACC, the majority of the literature consists of isolated case reports or small case series, and metastatic PCACC is even more infrequently encountered (18). To investigate the association between tumor diameter and metastasis in PCACC, we analyzed 74 cases with available data (non-metastasis: n = 55; metastasis: n = 19) (**Supplementary Material**). Among 74 cases with available tumor diameter and metastasis data, the metastasis group had a significantly larger median tumor diameter than the non-metastasis group (3.5 cm vs. 1.5 cm; P < 0.0001, Mann–Whitney U test) (**Figures 4A, B**). Receiver operating characteristic curve analysis of tumor diameter as a continuous predictor identified 2.0 cm as the optimal threshold by the Youden index (area under the curve, 0.817; sensitivity, 84.2%; specificity, 69.1%) (**Figure 4C**). In univariable logistic regression treating tumor diameter as a continuous variable, each 1-cm increase in tumor diameter was associated with higher odds of metastasis (OR, 1.93; 95% CI, 1.34–2.79; P = 0.0005; Wald test) (**Figure 4D**). In a separate dichotomous analysis, metastasis was documented in 16 of 33 patients (48.5%) with tumors ≥2.0 cm and in 3 of 41 patients (7.3%) with tumors <2.0 cm. The corresponding odds ratio for tumors ≥2.0 cm versus <2.0 cm was 11.92 (95% Wald CI, 3.06–46.42), and the association was significant by Fisher’s exact test (P < 0.0001). A positive point-biserial correlation was also observed between tumor diameter and metastatic status (r = 0.501, P < 0.0001). Descriptive subgroup analysis showed an overall increase in the observed frequency of metastasis with increasing tumor size: 0.0% (<1.0 cm), 8.8% (1.0–1.9 cm), 26.7% (2.0–2.9 cm), 75.0% (3.0–3.9 cm), and 64.3% (≥4.0 cm) (**Figure 4E**). Taken together, these findings support 2.0 cm as an exploratory threshold for identifying PCACC cases at increased risk of metastasis.

**Figure 4:**
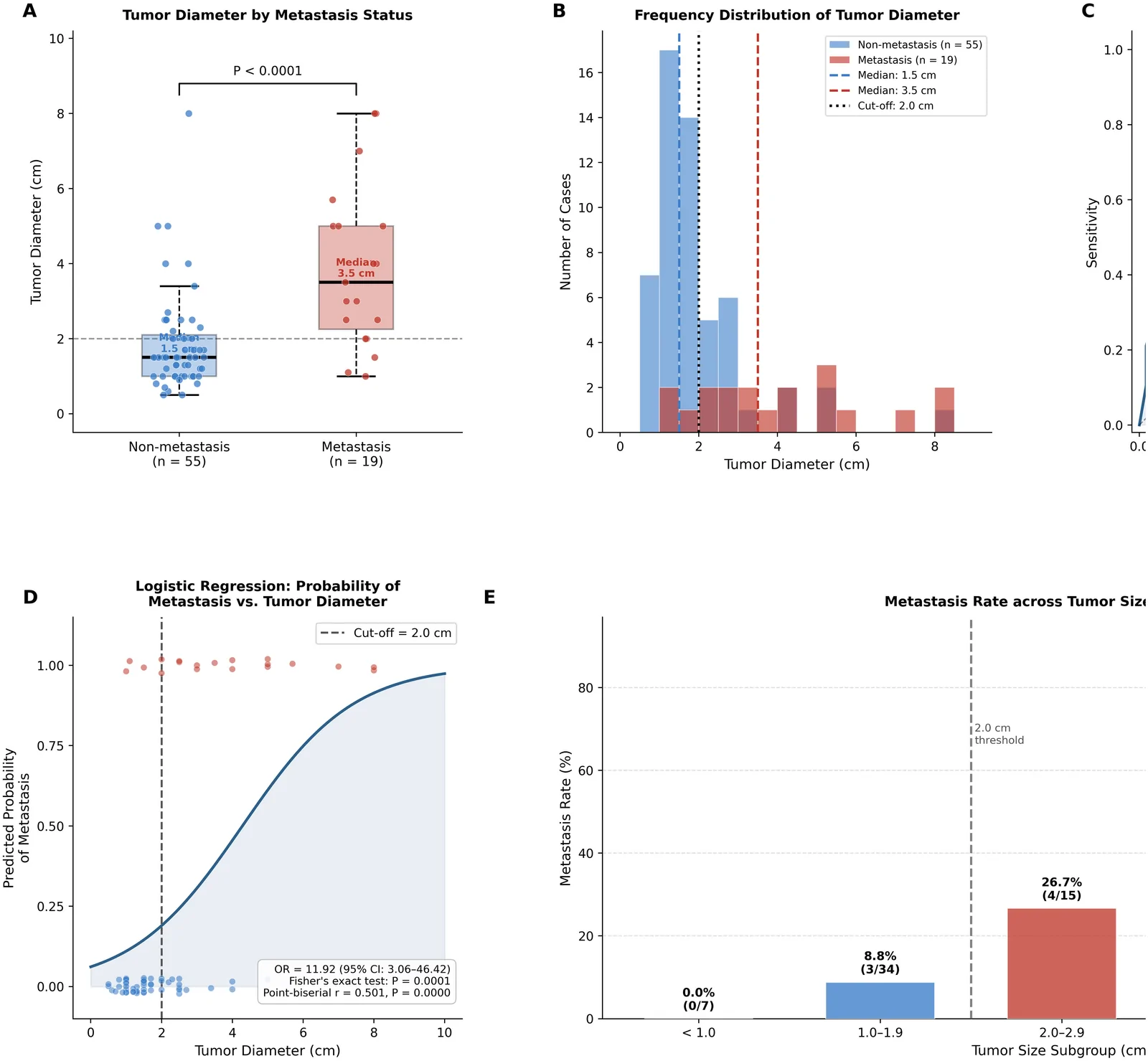
Correlation between tumor diameter and metastasis in primary cutaneous adenoid cystic carcinoma (PCACC). **(A)** Box-and-scatter plot comparing tumor diameters between the non-metastasis (n = 55) and metastasis (n = 19) groups. Median tumor diameter was significantly greater in the metastasis group than in the non-metastasis group (3.5 cm vs. 1.5 cm; P < 0.0001, Mann–Whitney U test). **(B)** Frequency distribution histogram of tumor diameters according to metastasis status. Dashed lines indicate the group-specific medians (blue, 1.5 cm; red, 3.5 cm), and the dotted black line denotes the 2.0 cm threshold. **(C)** Receiver operating characteristic (ROC) curve evaluating tumor diameter as a continuous predictor of metastasis (area under the curve, 0.817). The Youden index identified 2.0 cm as the optimal threshold, with a sensitivity of 84.2% and a specificity of 69.1%. **(D)** Logistic regression curve showing the predicted probability of metastasis according to tumor diameter modeled as a continuous variable. Each 1-cm increase in tumor diameter was associated with increased odds of metastasis (OR, 1.93; 95% CI, 1.34–2.79; P = 0.0005; Wald test). The vertical dashed line marks the 2.0 cm threshold. In a separate dichotomous analysis, metastasis was documented in 16 of 33 patients (48.5%) with tumors ≥2.0 cm and in 3 of 41 patients (7.3%) with tumors <2.0 cm. The corresponding sample OR was 11.92 (95% Wald CI, 3.06–46.42), and the association was significant by Fisher’s exact test (P < 0.0001). Tumor diameter was also positively correlated with metastatic status (point-biserial r = 0.501, P < 0.0001). **(E)** Bar chart showing the observed proportions of patients with documented metastasis across tumor size subgroups: <1.0 cm, 0.0% (0/7; 95% CI, 0.0%–41.0%); 1.0–1.9 cm, 8.8% (3/34; 95% CI, 1.9%–23.7%); 2.0–2.9 cm, 26.7% (4/15; 95% CI, 7.8%–55.1%); 3.0–3.9 cm, 75.0% (3/4; 95% CI, 19.4%–99.4%); and ≥4.0 cm, 64.3% (9/14; 95% CI, 35.1%–87.2%). The dashed line denotes the exploratory 2.0 cm threshold.

In conclusion, tumor diameter appears to be intimately associated with the biological behavior and metastatic potential of PCACC. Patients presenting with a tumor diameter of ≥ 2.0 cm may be considered as a higher-risk population. Given the potential for regional lymph node involvement, comprehensive staging imaging (e.g., whole-body PET-CT or thoracic/abdominal CT) could be considered for patients with larger tumors (≥2.0 cm) or those harboring additional high-risk histological features—such as subcutaneous adipose tissue infiltration, high-grade transformation, or extensive perineural invasion—to exclude occult metastatic disease. Additionally, sentinel lymph node biopsy (SLNB) might be considered on a case-by-case basis in this higher-risk cohort. For patients with clinically concerning or high-risk features, staging imaging may be considered on an individualized basis. The role of SLNB in PCACC remains investigational, and the present exploratory 2.0 cm candidate cut-off should not be used as an evidence-based indication for SLNB. Therapeutic lymph-node dissection should be reserved for clinically or radiologically confirmed nodal disease, in accordance with multidisciplinary assessment (19). Cutaneous adnexal malignancies, including PCACC, may clinically mimic benign cysts or other indolent nodules, thereby leading to an initial simple excision without oncologically adequate margins. In the present case, the innocuous and slowly enlarging lesion was initially managed as a presumed epidermal cyst, and margin involvement was recognized only on histopathological examination. Histopathological confirmation of PCACC should therefore prompt definitive re-excision with complete margin control to eradicate residual tumor and reduce the risk of local recurrence. Because metastasis in PCACC may manifest years or even decades after the initial resection, a more intensive and prolonged postoperative surveillance plan appears justified. Given the tumor’s inherent tendency for perineural invasion and distant spread, PCACC lesions exceeding 2.0 cm in diameter may warrant increased clinical vigilance and more comprehensive management by dermatologists and surgical dermatologists.

This study has several limitations. The pooled analysis was based on retrospectively extracted data from published case reports and small series, and is therefore susceptible to publication bias, selective reporting, incomplete clinical and pathological information, and historical variation in diagnostic criteria and treatment. Despite a structured duplicate-case assessment, unrecognized overlap between reports cannot be entirely excluded. Follow-up duration was heterogeneous, creating a risk of misclassifying patients with short follow-up as non-metastatic. Important prognostic covariates, including tumor depth, grade/high-grade transformation, perineural invasion, margin status, anatomical site, and treatment details, were incompletely reported and precluded robust multivariable modeling. Finally, the 2.0-cm cut-off was derived from and evaluated in the same small cohort without internal resampling or external validation. Accordingly, all size-related findings should be regarded as exploratory and hypothesis-generating rather than as evidence for a clinical decision threshold.

## Data Availability

The original contributions presented in the study are included in the article/**Supplementary Material**. Further inquiries can be directed to the corresponding author.
