# Supplementary material for: Primary cutaneous adenoid cystic carcinoma arising in the axilla: a case report and literature review
Source: Front Oncol. 2026 Sep 18;16:1942206. doi: 10.3389/fonc.2026.1942206 (PMC13631781; doi:10.3389/fonc.2026.1942206)
Supplement: Supplementary file 1 [file DataSheet1.docx]

**Supplementary Material2：**

**Method**

PubMed was searched from database inception to 30 April 2026 using the search term “Primary Cutaneous Adenoid Cystic Carcinoma”. Only English-language publications were considered. The initial search identified 157 records, all of which were assessed as full-text articles for eligibility. These articles contained 448 reported patient records. After assessment for potential overlap, 42 duplicate patients were excluded. Among the remaining unique patients, 332 were excluded because primary tumor size was not reported. Thus, 74 unique patients with available primary tumor-size and metastasis data were included in the exploratory statistical analysis, comprising 55 patients without documented metastasis during reported follow-up and 19 patients with documented nodal and/or distant metastasis.

**Supplementary Material：Summary of Published Cases of Primary Cutaneous Adenoid Cystic Carcinoma (PCACC)**

| **Year** | **Authors (reference)** | **Age（y）Sex** | **Primary site** | **Diameter (cm)** | **Recurrence** | **Metastasis** |
| --- | --- | --- | --- | --- | --- | --- |
| 1975 | Sanderson KV^[1]^ | 57F | scalp | 5.0 | Yes | lung |
| 1999 | Chang SE [2] | 70M | scalp | 2.5 | Yes | lung |
| 2001 | Chu SS [3] | 64M | left infra-auricular | 8.0 | Yes | neck lymph node |
| 2004 | Doganay L[4] | 55F | left axilla | 5.0 | Yes | axillary lymph node， lung |
| 2010 | Singh A[5] | 55M | infranasal | 5.0 | Yes | lung |
| 2013 | Ramakrishnan[6] | 71F | Scalp | 1.1 | Yes | regional lymph node |
| 2013 | Ramakrishnan[6] | 55F | Vulva | 3.0 | Yes | Yes |
| 2014 | Rocas D[7] | 47F | left breast | 1.5 | Yes | axillary lymph node |
| 2015 | Pozzobon LD[8] | 67M | left dorsal | 4.0 | Yes | axillary lymph node，lung，brain |
| 2016 | Pohl U[9] | 57F | left groin | 1.0 | Yes | lung, chest wall, lung，lymph node, brain |
| 2016 | Mohamed Ali AA[10] | 65F | left postauricular | 5.7 | Yes | Cervical lymph node, lung, bone |
| 2017 | Prieto-Granada CN[11] | 43M | right forearm | 7.0 | Yes | lymph node |
| 2017 | Singh GK[12] | 57M | right nasolabial fold | 2.0 | Yes | lung |
| 2022 | Lv JJ[13] | 54M | left forehead | 4.0 | Yes | brain |
| 2022 | Lv JJ[13] | 53F | scalp | 2.5 | Yes | lung, bone, shoulder |
| 2022 | Lv JJ[13] | 49F | scalp | 3.0 | Yes | lung, pleura |
| 2024 | El Mansoury FZ[14] | 79M | left hand | 8.0 | Yes | lung |
| 2025 | Geng X[15] | 57M | left ankle | 3.5 | NA | lung, bone, brain |
| 2025 | Kelsey E[16] | 60F | left thigh | 2.0 | NA | kidney, bone |
| 1986 | Lang PG[17] | 57M | scalp | 2.0 | NA | None |
| 1996 | Irvine AD[18] | 62M | scalp | 1.5 | NA | None |
| 2003 | Krunic AL[19] | 45M | scalp | 1.0 | NA | None |
| 2004 | Duzova AN[20] | 47F | scalp | 1.5 | NA | None |
| 2008 | Barnes J[21] | 62F | scalp | 1.5 | NA | None |
| 2008 | Naylor E[22] | 85F | scalp | 1.5 | NA | None |
| 2008 | Naylor E[22] | 62M | scalp | 1.0 | NA | None |
| 2008 | Numajiri T[23] | 56M | perineum | 8.0 | NA | None |
| 2009 | Cansu DU[24] | 54M | back | 1.5 | NA | None |
| 2010 | Torres T[25] | 55F | scalp | 1.5 | NA | None |
| 2010 | Xu YG[26] | 58M | scalp | 1.5 | NA | None |
| 2011 | Cacchi C[27] | 28F | abdomen | 1.0 | NA | None |
| 2012 | Raychaudhuri S[28] | 31M | chest wall | 1.3 | NA | None |
| 2013 | Maybury CM[29] | 58F | groin | 1.7 | NA | None |
| 2013 | Ramakrishnan[6] | 62F | Temporo-parietal region | 1.3 | None | None |
| 2013 | Ramakrishnan[6] | 75M | Left knee | 1.7 | Yes, | None |
| 2013 | Ramakrishnan[6] | 72F | Right forearm | 1.3 | Yes | None |
| 2013 | Ramakrishnan[6] | 70M | Right groin | 2.5 | Yes | None |
| 2013 | Ramakrishnan[6] | 70M | Scalp | 4.0 | Yes | None |
| 2013 | Ramakrishnan[6] | 46M | Left arm | 2.0 | Yes | None |
| 2013 | Ramakrishnan[6] | 81M | Right leg | 2.5 | None | None |
| 2013 | Ramakrishnan[6] | 77M | Scalp | 5.0 | Yes | None |
| 2013 | Ramakrishnan[6] | 58M | Axilla | 1.5 | None | None |
| 2013 | Ramakrishnan[6] | 60F | Neck | 2.5 | None | None |
| 2013 | Ramakrishnan[6] | 28F | Right Cheek | 2.7 | None | None |
| 2013 | Ramakrishnan[6] | 54M | Lumbar | 1.7 | None | None |
| 2013 | Ramakrishnan[6] | 31M | Lumbar | 1.5 | Yes | None |
| 2013 | Ramakrishnan[6] | 51M | Vertex Scalp | 1.0 | None | None |
| 2013 | Ramakrishnan[6] | 72M | Right temporo-parietal region | 2.0 | None | None |
| 2013 | Ramakrishnan[6] | 62F | Midline-parietal region | 2.2 | NA | None |
| 2013 | Ramakrishnan[6] | 78F | Scalp | 5.0 | None | None |
| 2013 | Ramakrishnan[6] | 75M | Neck | 2.5 | None | None |
| 2013 | Ramakrishnan[6] | 74M | Scalp | 4 | None | None |
| 2013 | Ramakrishnan[6] | 62F | Temporo-parietal region | 1.3 | None | None |
| 2013 | Ramakrishnan[6] | 75M | Left knee | 1.7 | Yes | None |
| 2014 | Morrison AO[30] | 62F | scalp | 0.6 | NA | None |
| 2015 | Matsumoto N[31] | 65M | scalp | 3.4 | NA | None |
| 2015 | Nakajima H[32] | 65M | scalp | 1.0 | NA | None |
| 2016 | Lee SJ[33] | 66M | umbilicus | 1.0 | NA | None |
| 2017 | Prieto-Granada[11] | 55M | left scalp | 0.7 | None | None |
| 2022 | Lv JJ[13] | 79M | left buttock | 1.0 | Yes | None |
| 2022 | Lv JJ[13] | 41F | chest | 0.8 | None | None |
| 2022 | Lv JJ[13] | 50M | left face | 1.0 | None | None |
| 2022 | Lv JJ[13] | 79M | abdomen | 0.8 | None | None |
| 2022 | Lv JJ[13] | 53M | left lower limb | 1.2 | None | None |
| 2022 | Lv JJ[13] | 58F | abdomen | 0.5 | None | None |
| 2022 | Lv JJ[13] | 45M | left axilla | 0.9 | None | None |
| 2022 | Lv JJ[13] | 67M | forehead | 2.3 | None | None |
| 2022 | Lv JJ[13] | 64F | scalp | 1.2 | None | None |
| 2022 | Lv JJ[13] | 46M | right chest | 1.0 | None | None |
| 2022 | Lv JJ[13] | 53F | head and neck/trunk | 1.0 | NA | None |
| 2022 | Temnithikul B[34] | 70F | scalp | 0.5 | NA | None |
| 2024 | Soughi M[35] | 66F | scalp | 2.5 | NA | None |
| 2025 | Mokhtar J[36] | 50F | scalp | 1.2 | NA | None |
| 2026 | Hu SS | 37F | right axilla | 1.5 | Yes | None |

# References

[1]	Sanderson KV, Batten JC. Adenoid cystic carcinoma of the scalp with pulmonary metastasis. Proc R Soc Med. 1975;68; 649–650.

[2]	Chang SE, Ahn SJ, Choi JH, Sung KJ, Moon KC, Koh JK. Primary adenoid cystic carcinoma of skin with lung metastasis. J Am Acad Dermatol. United States, 1999. 640–642.

[3]	Chu SS, Chang YL, Lou PJ. Primary cutaneous adenoid cystic carcinoma with regional lymph node metastasis. J Laryngol Otol. 2001;115; 673–675.

[4]	Doganay L, Bilgi S, Aygit C, Altaner S. Primary cutaneous adenoid cystic carcinoma with lung and lymph node metastases. J Eur Acad Dermatol Venereol. England, 2004. 383–385.

[5]	Singh A, Ramesh V. Primary cutaneous adenoid cystic carcinoma with distant metastasis: a case report and brief literature review. Indian J Dermatol Venereol Leprol. United States, 2010. 176–179.

[6]	Ramakrishnan R, Chaudhry IH, Ramdial P, Lazar AJ, McMenamin ME, Kazakov D, et al. Primary cutaneous adenoid cystic carcinoma: a clinicopathologic and immunohistochemical study of 27 cases. Am J Surg Pathol. United States, 2013. 1603–1611.

[7]	Rocas D, Asvesti C, Tsega A, Katafygiotis P, Kanitakis J. Primary adenoid cystic carcinoma of the skin metastatic to the lymph nodes: immunohistochemical study of a new case and literature review. Am J Dermatopathol. 2014;36; 223–228.

[8]	Pozzobon LD, Glikstein R, Laurie SA, Hanagandi P, Michaud J, Purgina B, et al. Primary cutaneous adenoid cystic carcinoma with brain metastases: case report and literature review. J Cutan Pathol. 2016;43; 137–141.

[9]	Pohl U, Said I, Low HL. Rare delayed brain metastasis from primary cutaneous adenoid cystic carcinoma. Clin Neuropathol. Germany, 2016. 39–41.

[10]	Mohamed Ali AA, Sharma P, Rege R, Seena C, Kulasekaran N, Rajesh S. CT and MR of recurrent primary cutaneous adenoid cystic carcinoma with multiple metastases. BJR Case Rep. 2016;2; 20150243.

[11]	Prieto-Granada CN, Zhang L, Antonescu CR, Henneberry JM, Messina JL. Primary cutaneous adenoid cystic carcinoma with MYB aberrations: report of three cases and comprehensive review of the literature. J Cutan Pathol. 2017;44; 201–209.

[12]	Singh GK, Singh P, Kaur J, Kumar R. Lung metastasis in primary cutaneous adenoid cystic carcinoma - Clinicopathological evaluation of a rare case with review of literature. J Egypt Natl Canc Inst. 2017;29; 163–165.

[13]	Lv JJ, Ren M, Cai X, Hu J, Kong JC, Kong YY. Primary cutaneous adenoid cystic carcinoma: a clinicopathologic, immunohistochemical, and fluorescence in-situ hybridisation study of 13 cases. Histopathology. 2022;80; 407–419.

[14]	El Mansoury FZ, Messaoud O, Essaber H, Allioui S, El Bakkari A, Jerguigue H, et al. Primary cutaneous adenoid cystic carcinoma with lung metastasis: A case report with literature review. Radiol Case Rep. Netherlands, 2024. 617–620.

[15]	Geng X, Sun D, Sun H, Zhang C, Li J, Hao F, et al. Multiline treatment of primary cutaneous adenoid cystic carcinoma with multiple metastases: a case report and literature review. Front Oncol. 2025;15; 1669171.

[16]	Kelsey E, Broad A, Magree C, Grills R. Primary cutaneous adenoid cystic carcinoma metastasising to the kidney. BMJ Case Rep. 2025;18.

[17]	Lang PG, Jr., Metcalf JS, Maize JC. Recurrent adenoid cystic carcinoma of the skin managed by microscopically controlled surgery (Mohs surgery). J Dermatol Surg Oncol. 1986;12; 395–398.

[18]	Irvine AD, Kenny B, Walsh MY, Burrows D. Primary cutaneous adenoid cystic carcinoma. Clin Exp Dermatol. 1996;21; 249–250.

[19]	Krunic AL, Kim S, Medenica M, Laumann AE, Soltani K, Shaw JC. Recurrent adenoid cystic carcinoma of the scalp treated with mohs micrographic surgery. Dermatol Surg. United States, 2003. 647–649.

[20]	Duzova AN, Boztepe G, Sahin S, Gokoz A. Painful nodule on the scalp: a case of primary cutaneous adenoid cystic carcinoma. Acta Derm Venereol. 2004;84; 243–244.

[21]	Barnes J, Garcia C. Primary cutaneous adenoid cystic carcinoma: a case report and review of the literature. Cutis. 2008;81; 243–246.

[22]	Naylor E, Sarkar P, Perlis CS, Giri D, Gnepp DR, Robinson-Bostom L. Primary cutaneous adenoid cystic carcinoma. J Am Acad Dermatol. United States, 2008. 636–641.

[23]	Numajiri T, Nishino K, Uenaka M. Giant primary cutaneous adenoid cystic carcinoma of the perineum: histological and radiological correlations. Acta Derm Venereol. 2008;88; 316–318.

[24]	Cansu DU, Kasifoglu T, Ackaln M, Korkmaz C. The development of primary cutaneous adenoid cystic carcinoma in a patient with ankylosing spondylitis treated with etanercept. South Med J. 2009;102; 738–740.

[25]	Torres T, Caetano M, Alves R, Horta M, Selores M. Tender tumor of the scalp: clinicopathologic challenge. Primary cutaneous adenoid cystic carcinoma. Int J Dermatol. England, 2010. 605–607.

[26]	Xu YG, Hinshaw M, Longley BJ, Ilyas H, Snow SN. Cutaneous adenoid cystic carcinoma with perineural invasion treated by mohs micrographic surgery-a case report with literature review. J Oncol. 2010;2010; 469049.

[27]	Cacchi C, Persechino S, Fidanza L, Bartolazzi A. A primary cutaneous adenoid-cystic carcinoma in a young woman. Differential diagnosis and clinical implications. Rare Tumors. England, 2011. e3.

[28]	Raychaudhuri S, Santosh KV, Satish Babu HV. Primary cutaneous adenoid cystic carcinoma of the chest wall: a rare entity. J Cancer Res Ther. India, 2012. 633–635.

[29]	Maybury CM, Patel K, Attard N, Robson A. A nodule in the groin. Primary cutaneous adenoid cystic carcinoma (pcACC). JAMA Dermatol. United States, 2013. 1343–1344.

[30]	Bui M, Frank SJ, Nasser QJ, El Sawy T, McLemore MS, Morrison WH, et al. Multidisciplinary management of primary adenoid cystic carcinoma of the eyelid with perineural invasion. Ophthalmic Plast Reconstr Surg. 2013;29; e143–146.

[31]	Matsumoto N, Hata Y, Tanese K. Case of primary cutaneous adenoid cystic carcinoma: Expression of c-KIT and activation of its downstream signaling molecules. J Dermatol. 2015;42; 1109–1111.

[32]	Nakajima H, Matsushima T, Sano S. Primary cutaneous adenoid cystic carcinoma arising from folliculitis decalvans. J Dermatol. 2015;42; 741–743.

[33]	Lee SJ, Yang WI, Kim SK. Primary Cutaneous Adenoid Cystic Carcinoma Arising in Umbilicus. J Pathol Transl Med. Korea (South), 2016. 322–324.

[34]	Temnithikul B, Rungrunanghiranya S, Limtanyakul P, Jerasuthat S, Paige DG. Primary cutaneous adenoid cystic carcinoma of the scalp: A case report, immunohistochemistry and review of the literature. Skin Health Dis. England, 2022. e118.

[35]	Soughi M, Elloudi S, Ardigo M, Baybay H, Mernissi F. Primary Cutaneous Adenoid Cystic Carcinoma: A Misdiagnosed Tumor. Cureus. 2024;16; e64318.

[36]	Mokhtar J, Akbarpoor F, Oghanna NG, Bennett NJ. Primary cutaneous adenoid cystic carcinoma arising in the scalp: a diagnostic challenge. BMJ Case Rep. England, 2025.
